# Incorporation of information diffusion model for enhancing analyses in HIV molecular surveillance

**DOI:** 10.1080/22221751.2020.1718554

**Published:** 2020-01-30

**Authors:** Tsz Ho Kwan, Ngai Sze Wong, Grace Chung Yan Lui, Kenny Chi Wai Chan, Owen Tak Yin Tsang, Wai Shing Leung, Kai Man Ho, Man Po Lee, Wilson Lam, Sze Nga Chan, Denise Pui Chung Chan, Shui Shan Lee

**Affiliations:** a Jockey Club School of Public Health and Primary Care, The Chinese University of Hong Kong, Shatin, Hong Kong; b Stanley Ho Centre for Emerging Infectious Diseases, The Chinese University of Hong Kong, Shatin, Hong Kong; c Department of Medicine and Therapeutics, The Chinese University of Hong Kong, Shatin, Hong Kong; d Integrated Treatment Centre, Department of Health, Kowloon Bay, Hong Kong; e Department of Medicine and Geriatrics, Princess Margaret Hospital, Lai Chi Kok, Hong Kong; f Department of Medicine, Queen Elizabeth Hospital, Kowloon, Hong Kong

**Keywords:** Molecular epidemiology, HIV, information diffusion, network analysis, men who have sex with men

## Abstract

Molecular surveillance of infections is essential in monitoring their transmission in the population. In this study, newly diagnosed HIV patients' phylogenetic, clinical and behavioural data were integrated, and an information diffusion model was incorporated in analysing transmission dynamics. A genetic network was constructed from HIV sequences, from which transmission cascades were extracted. From the transmission cascades, CRF01_AE had higher values of information diffusion metrics, including scale, speed and range, than that of B, signifying the distinct transmission patterns of two circulating subtypes in Hong Kong. Patients connected in the network, were more likely male, younger, of main circulating subtypes, to have acquired HIV infection locally, and a higher CD4 level at diagnosis. Genetic connections varied among men who have sex with men (MSM) who used different channels of sex networking and varied in their engagement in risk behaviours. MSM using recreational drugs for sex held positions of greater importance within the network. Significant differences in network metrics were observed among MSM as differentiated by their mobile apps usage patterns, evidencing the impact of social network on transmission networks. The applied model in the presence of consistently collected longitudinal data could enhance HIV molecular epidemiologic surveillance for informing future intervention planning.

## Introduction

As for other infectious diseases, molecular surveillance of HIV infections commonly encompasses phylogeny-based analysis of sequence data derived from the circulating virus. Coalescent theory is often implied for interpreting the genealogies. However, in the presence of multiple exogenous sources of infection or the emergence of recombinant strains, the assumption in coalescent theory is often violated. In such circumstances, a network approach can be more applicable as it does not assume all sequences to be descendants from one common ancestor but rather compares the dissimilarity amongst them [1]. Such genetic network-based analysis is particularly relevant for examining HIV transmission dynamics in the population where multiple subtypes were in circulation [2–4]. As a predominantly sexually transmitted infection, HIV forms networks which do not occur randomly but are behaviourally driven and shaped by treatment interventions [5–6]. Proper integration of behavioural and clinical attributes is essential for constructing models to support the interpretation of the transmission dynamics of HIV in the population [2,6–7].

We reckon that HIV transmission in the community is analogous to that of information diffusion in a social network [8]. The paths on which information is passed on in the network form multiple information cascades. How information spreads through the network depends on the node’s “infectiousness,” “resistance” and network topology [9–11]. The mechanism of information diffusion process can be viewed as a symmetric push–pull activity [12]. The strength of the push process could be measured by “infectiousness” expressed as the number of nodes the sender could “infect” (scale) and the average number of “infections” within a given time interval (speed) [13]. Some actors in the network may be more “infectious” in the diffusion of information, such as those connected to a large number of nodes or are in multiple information cascades. These nodes often have a high degree or betweenness centrality. How well a node receives a message in the pull process is a function of “resistance.” The topology of network plays an important role in information diffusion. In general, the clusteredness of the network is a surrogate of how easy information could be delivered to each node in the network. In describing HIV transmission, the “infectiousness” of information is likened to virus infectivity, “resistance” reflects the protective means, if any, adopted by an individual. Topology’s role in HIV transmission is likewise important. Isolated HIV in small clusters are difficult to reach and their ability to pass on would be limited compared to a large clustered network, similar to the situation for information diffusion. A dense network also allows information, or a virus, to be transmitted at fewer steps, therefore the range of network or the level of information cascade would be lower.

In this study, the information diffusion model was incorporated in characterizing real-world HIV molecular epidemiology in Hong Kong, a metropolitan city where sexual transmission is the main mode of virus spread. Clinical history and behavioural profiles were simultaneously collected from newly diagnosed HIV patients whose viruses were sequenced to contribute to the integrated analyses for enhancing molecular surveillance.

## Material and methods

### Study design

Over a 2-year period, a cohort study was conducted to recruit newly diagnosed patients in Hong Kong from all four HIV specialist clinics. Eligibility criteria included: age 18 years or above, able to communicate in written and spoken Chinese or English, and being treatment-naive. Prisoners and patients with mental illnesses who were unable to make an informed decision were excluded. With consent, participants were asked to complete a self-administered questionnaire and have blood samples collected for HIV sequencing. Their clinical data were transcribed from matched medical records the following consent. The study was approved by The Joint Chinese University of Hong Kong – New Territories East Cluster Clinical Research Ethics Committee (Ref. No.: CREC2015.232). Institutional approval was also obtained from the collaborating sites where patients were recruited: Queen Elizabeth Hospital, Princess Margaret Hospital, Prince of Wales Hospital, and Integrated Treatment Centre of Department of Health.

### Data collection

#### Behavioural questionnaire

The questionnaire consisted of circumstances of diagnosis and infection, behavioural profile history during lifetime and one year before infection, and demographics. Diagnosis year, month, and location were enquired. Participants were asked to estimate location, time, and source of infection. Behavioural profiles were divided in accordance with one’s HIV exposure history(ies): men who had sex with men (MSM), men who had sex with women, women who had sex with men, and injection drug users (IDU). A bisexual IDU would need to complete three parts. In the sex-related questionnaires, sex networking patterns including channels and frequency, condom use and drug use in the context of facilitating sex (chemsex), and sexually transmitted infection (STI) infection history were enquired. Patients who had ever injected drugs were asked about their drug and methadone use, as well as injection-related networking patterns. Selected demographic variables, viz., age and sex, behavioural and networking patterns among MSM were parametrized in subsequent network analyses.

#### Clinical data

Route of transmission, antiretroviral treatment (ART) regimen and its start date, longitudinal CD4 and viral load measures were transcribed from participants’ clinical records.

#### HIV sequence

HIV RNA was extracted from blood samples and the drug resistance test position of pol gene was sequenced using Sanger sequencing as described previously [5]. HIV subtyping was performed using REGA HIV-1 Subtyping Tool [14].

### Analysis

**Figure 1.**
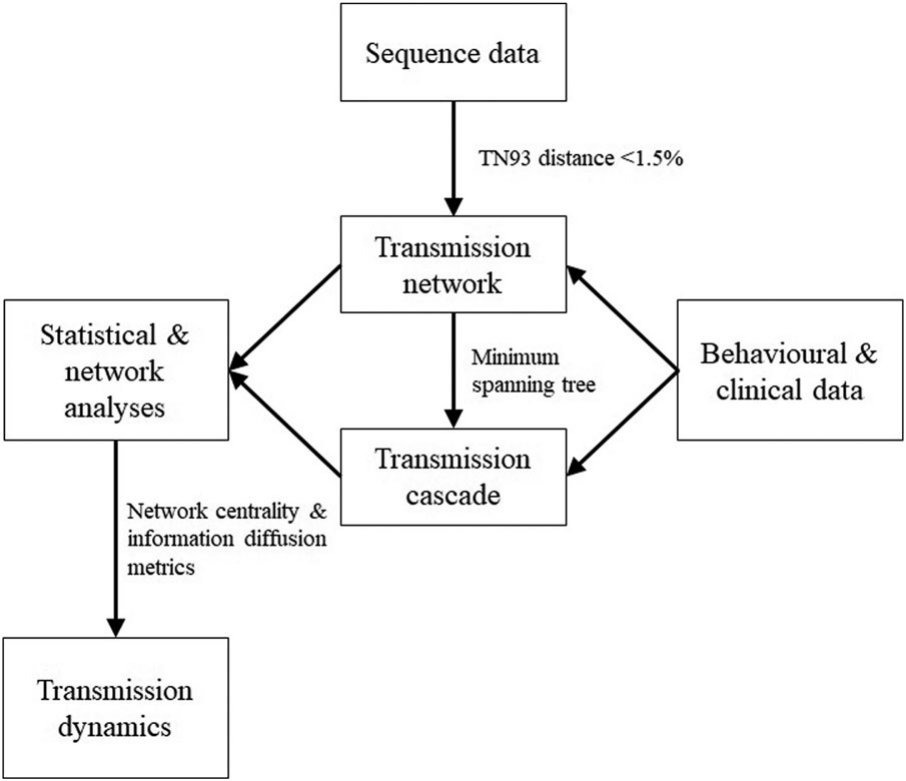
Analytical framework of the study.

Patients with viral sequences were included in the analyses. Sequences were assembled and aligned before TN93 pairwise distance was calculated. Pairs with less than 1.5% distance were used to construct a genetic network. Each genetic cluster was described by network metrics, including the number of nodes and edges, and density. Duration of transmission potential was calculated by the difference between the latest diagnosis year and the earliest infection year plus one. To assess the importance of a node, degree and betweenness centrality were calculated by the number of edges, and proportion of shortest paths passing through the node, respectively. Comparison was made between patients in the genetic network and those who were not, using logistic regression and the Wilcoxon rank sum test. To extract transmission cascades, undirected transmission clusters became directed by incorporating the order of self-reported infection time, an approach adopted by a previous study [15]. Bidirectional edges were formed between nodes with unknown infection time or in the same month. The directionality of edges does not imply transmission relationship, but the transmission history along the cascade. There can be intermediate nodes between two sampled nodes while the infection history would be unchanged. The minimum spanning tree of each transmission cluster was extracted as a transmission cascade by Prim’s algorithm with genetic distance as weight [16]. The analytical framework of the study is summarized in Figure 1.

The following information diffusion metrics were measured: scale, range and speed. Scale of a transmission cascade was defined by its highest node degree; its range referred to the longest directed path along the cascade; and its speed defined by the number of nodes in the cascade divided by the duration of transmission potential. The metrics were compared between subtypes. All analyses were conducted using R (R Foundation for Statistical Computing, Austria).

## Results

Between 2016 and 2018, a total of 438 newly diagnosed HIV patients with sequence data were recruited. A majority of the recruited subjects (384, 88%) were MSM, 49 (11%) had acquired infection through heterosexual contacts, 2 were IDU and the remaining 3 did not specify their routes of transmission (Table 1). The median year of birth was 1985 (interquartile range: 1975–1991). The most prevalent subtypes were B (33%), CRF01_AE (33%) and CRF07_BC (14%). Some 14% were unique recombinant forms (URFs). The remaining 6% were subtypes CRF02_AG, CRF08_BC, A, C and G.

**Table 1.** Comparison between connected (*n* = 185) and unconnected (*n* = 253) sequences in the transmission network.

Variable	Category	Total *n* (%) *Median (IQR)*	Connected in the transmission network *n* (%) *Median (IQR)*	Unconnected in the transmission network n (%) *Median (IQR)*	Odds ratio (95% confidence interval) *W*	*p*
Gender	Female	17 (4%)	2 (1%)	15 (6%)	1	–
	Male	421 (96%)	179 (99%)	242 (94%)	5.55 (1.25–24.57)	.01
Year of birth	–	*1985* (*1975–1991)*	*1987* (*1978–1993)*	*1982* (*1972–1989)*	*17643*	<.001
Mode of transmission	Non-MSM	54 (12%)	10 (6%)	43 (17%)	1	–
	MSM	384 (88%)	171 (94%)	213 (83%)	3.45 (1.69–7.07)	<.001
HIV subtype	CRF01_AE	145 (33%)	70 (39%)	75 (29%)	1	–
	B	145 (33%)	76 (42%)	69 (27%)	1.18 (0.74–1.87)	.48
	CRF07_BC	61 (14%)	13 (7%)	48 (19%)	0.29 (0.15-–0.58)	<.001
	Others	87 (20%)	22 (12%)	65 (25%)	0.36 (0.20–0.65)	.001
Place of infection	Hong Kong	323 (74%)	159 (90%)	164 (65%)	1	–
	Mainland China	51 (12%)	7 (4%)	44 (18%)	0.16 (0.07–0.38)	<.001
	Other	64 (15%)	15 (8%)	49 (19%)	0.32 (0.17–0.59)	<.001
Infection year	–	*2016* (*2016–2017)*	*2016* (*2016–2017)*	*2016* (*2016–2017)*	*20399*	.26
Diagnosis year	–	*2017* (*2016–2017)*	*2017* (*2016–2017)*	*2017* (*2017–2017)*	*20366*	.14
CD4 at diagnosis	–	*313* (*193–436)*	*336* (*221–446)*	*288* (*170–412)*	*20021*	.01
Viral load at diagnosis	–	*4.83* (*4.28–5.32)*	*4.71* (*4.26–5.30)*	*4.91* (*4.28–5.36)*	*24679*	.19

Notes: IQR interquartile range; MSM men who have sex with men.

### Formation of genetic networks

**Figure 2.**
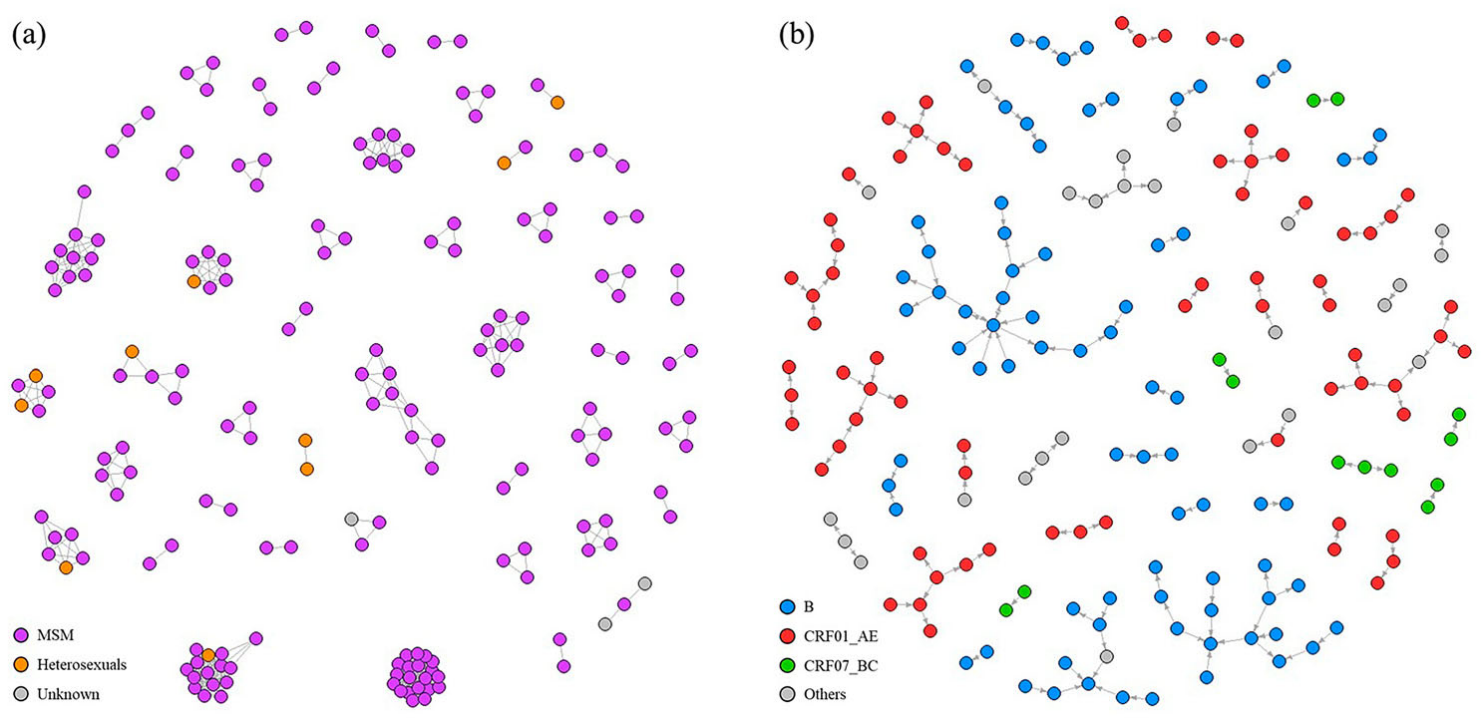
Genetic network with route of transmission (a) and transmission cascades with subtype (b) of HIV sequences (*n* = 185).

Using a genetic distance threshold of 1.5%, 491 links were established between 185 (42%) sequences (Figure 2(a)). Of 47 clusters, 20 (43%) were dyads. The largest cluster contained 20 nodes. Subtype CRF01_AE clusters contributed to 40%, while 30% and 6% were subtype B and CRF07_BC clusters, respectively. Clusters with the latest infection year before 2018 had fewer nodes (*p* = .02). Particularly, dyads had a shorter period of transmission potential (median 1 year vs 2.5 years, *p* = .01).

Comparison was made between patients connected within genetic networks (*n* = 185) and those unconnected (*n* = 253). Patients in the genetic network were more likely male (odds ratio [OR] 5.55, 95% confidence interval [CI] 1.25–24.57, *p* = .01), MSM (OR 3.45, 95% CI 1.69–7.07, *p* < .001) and had acquired HIV infection locally, i.e. within the territory of Hong Kong (*p* < .001) (Table 1). Patients of subtype CRF01_AE and B were more likely to be connected than other subtypes or URFs (*p* < .001). Of all URFs identified in this study (*n* = 63), no characteristic connectivity patterns could be delineated. CRF07_BC cascades were observed as a collection of dyads. Younger age (*p* < .001) and higher CD4 at diagnosis (*p* = .01) were also positively associated with having genetic connections. Years of diagnosis and infection, and viral load at diagnosis were not different between connected and unconnected.

### Sex networking and genetic networks

The sex-networking patterns of MSM before HIV diagnoses were examined and correlated with genetic connectedness. The main channels of sex-networking were: use of mobile apps (72%), and frequenting saunas (47%), bars (32%), public toilets (11%) and beaches (7%) (Table 2). Younger MSM (*p* < .001), those infected locally (*p* < .001), use of mobile apps for sex networking (*p* = .004), and engagement in chemsex (*p* = .01) were associated with being connected in the genetic network. MSM who visited saunas (*p* = .03) or public toilets (*p* = .045) for sex networking were less likely to be connected. MSM with genetic links had a higher CD4 cell count at diagnosis (*p* = .04). MSM who used an international gay app for partner sourcing (median 0.00, IQR 0.00–0.01 vs. 0.00, 0.00–0.00, *p* = .01) and had engaged in chemsex (median 0.00, IQR 0.00–0.02 vs. 0.00, 0.00–0.001, *p* = .01) a year before infection had a higher betweenness centrality. MSM who had used a gay app which was popular in mainland China (median 1.00, IQR 1.00–2.75 vs. 3.00, 2.00–6.00, *p* = .03) and visited spa for sex (median 2.00, IQR 1.00–4.75 vs 3.00, 2.00–6.00, *p* = .01) a year before infection had a lower degree centrality.

**Table 2.** Comparison between men who have sex with men (MSM) who were connected and unconnected in the transmission network (*N* = 384).

Variable	Total n (%) *Median (IQR)*	Connected in the transmission network n (%) *Median (IQR)*	Unconnected in the transmission network n (%) *Median (IQR)*	Odds ratio (95% confidence interval) *W*	*p*
Year of birth	*1986* (*1978–1992)*	*1988* (*1980–1993)*	*1976* (*1984–1990)*	*14460*	.001
Local infection (HIV transmission within Hong Kong)	302 (79%)	154 (90%)	148 (69%)	3.98 (2.23–7.10)	<.001
Infection year	*2016* (*2016–2017)*	*2016* (*2016–2017)*	*2016* (*2016–2017)*	*16579*	.30
Favoured sex networking channels before HIV diagnosis (*N* = 378)					
• Bars	121 (32%)	45 (27%)	76 (36%)	0.67 (0.43–1.04)	.07
• Saunas	176 (47%)	67 (40%)	109 (51%)	0.64 (0.42–0.96)	.03
• Public toilets	41 (11%)	12 (7%)	29 (14%)	0.49 (0.24–0.996)	.045
• Beaches	25 (7%)	13 (8%)	12 (6%)	1.42 (0.63–3.19)	.40
• Mobile apps	274 (72%)	129 (78%)	145 (68%)	1.61 (1.01–2.57)	.04
In the year before infection					
• Used an international gay mobile app for sex networking (*N* = 336)	226 (67%)	116 (76%)	110 (60%)	2.17 (1.35–3.49)	.001
• Used a mainland Chinese gay mobile app for sex networking (*N* = 336)	33 (10%)	6 (4%)	27 (15%)	0.24 (0.10–0.59)	.001
• Engaged in chemsex (*N* = 371)	227 (61%)	76 (47%)	68 (33%)	1.80 (1.18–2.74)	.01
Diagnosis year	2017 (2016–2017)	2016 (2017–2017)	2017 (2017–2017)	*19082*	.09
CD4 at diagnosis	319 (209–437)	338 (224–448)	300 (187–410)	*16035*	.04
Log viral load at diagnosis	*4.83* (*4.30–5.34)*	*4.71* (*4.29–5.30)*	*4.91* (*4.33–5.41)*	*19499*	.15

Note: IQR interquartile range.

### Information diffusion metrics of transmission cascades

Transmission cascades had a median scale of 2.00 (IQR 1.00–2.00), a median range of 1.00 (IQR 1.00–2.00) and a median speed of 1.00 (IQR 0.67–1.33) (Figure 2(b)). The three largest cascades were composed of subtype B sequences, which also gave the highest scale among all transmission cascades. Seven out of 16 (44%) subtype B cascades were dyads, while only 4 (20%, *N* = 20) subtype CRF01_AE cascades were dyads. Cascades of subtype CRF01_AE had higher scale (*p* = .03, median 2.0, IQR 1.75–3.00 vs 1.00, 1.00–2.00), speed (*p* = .047, median 1.00, IQR 0.92–2.00 vs 0.67, 0.67–1.00) and range (*p* = .049, median 1.50, IQR 1.00–2.00 vs 1.00, 1.00–1.00) compared to non-CRF01_AE cascades.

## Discussion

Our study shows that in Hong Kong, different subtypes of HIV have been circulating in the population forming multiple lineages. Through an integration of clinical and behavioural data coupled with molecular analyses, we have demonstrated that locally infected persons and MSM were more likely to be connected in the transmission networks, confirming that the HIV epidemic, particularly that of subtypes CRF01_AE and B, was driven by local transmission among MSM, especially the younger ones. CRF07_BC was less likely to be connected within Hong Kong’s local networks. As one of the signature virus subtypes in China, their transmission may be related to infection sources from mainland China [17], corresponding to our finding that HIV-positive MSM who used a mainland Chinese gay mobile app were also less likely to be connected. Healthier HIV infected MSM, as reflected from a higher CD4 count at diagnosis, may be unaware of his infection and could have maintained their activity in sex networking thereby supporting ongoing transmission [18–19]. The higher odds of connectivity among younger MSM suggested age-assortativity and they may link the infections between younger and older MSM [20]. Engaging in chemsex was identified as a factor for connectivity and its high betweenness centrality suggested that such activity could form a hub of transmission. Chemsex often took place in private premises and the participants were often recruited online, especially through mobile apps [21]. On the other hand, public venue goers frequenting sauna, public toilet and spa had a lower connectivity in the network, as compared to the observed higher betweenness centrality among app users. MSM using mobile apps for sex networking had a higher prevalence of condomless anal sex [22], which predisposed them to the risk of HIV infection and therefore a higher connectivity within the HIV genetic network. Transmission clusters were smaller if the latest infection year was earlier, which could either be a result of ART that prevented onward transmission, or those infected later had not been diagnosed yet. The shorter infection to diagnosis time observed in dyads could be attributed to more frequent HIV testing such that the transmission chain ended, or a result of missing nodes [23].

Insights into the HIV transmission dynamics in Hong Kong were revealed by the adaption of the information diffusion model. While subtype B and CRF01_AE were separately shown to be connected in networks, their diffusion patterns varied. Some complex, large cascades were found in subtype B, yet the majority were sequential chains which averaged out size-sensitive information diffusion metrics when comparing with subtype CRF01_AE whose cascades were primarily medium-sized. The longer range in subtype CRF01_AE cascades suggested that virus transmission had occurred along the cascade one by one. Hypothetically a cascade of the same size but the lower range would have a larger scale, signifying the occurrence of one-to-many transmission or the transmission had occurred within a short period of time. Subtype CRF01_AE was not the dominant virus among MSM in Hong Kong [24] but from the results, we noticed its transmission had gained momentum in the past few years. The three large subtype B cascades continued to grow locally during the study period; meanwhile, there appears to be a risk of sporadic import of CRF07_BC strains which may circulate locally if they remain active in the transmission cascades.

Recalling information diffusion as a symmetric push–pull activity, the transmission could not have happened in the absence of either. In planning intervention strategies, removing inward (pull strategy) or outward (push strategy) edges carries a similar blocking effect for preventing onward HIV transmissions. ART currently plays a key role in halting HIV transmission as both a push and a pull strategy. For an HIV-positive node, its infectiousness would be minimized or even eliminated if ART is given and well-adhered to [25]. If viral suppression has been achieved, the node would lose its ability to “push” the infection to downstream nodes. On the other hand, pre-exposure prophylaxis (PrEP) with antiretrovirals deactivates the pull strategy that reduces the risk of HIV acquisition in an uninfected person (node) [26]. Similar to herd immunity, HIV spread could be contained if potential transmitters are protected as a priority. Our results showed that chemsex activity played an important role in sustaining transmission chains, and younger MSM who sought sex partners using mobile apps driving the local HIV epidemic should be targeted for promoting and delivering PrEP and other HIV prevention materials.

This study carries several limitations. First, similar to other genetic network studies, linkages between patients were inferred from their viral genetic similarity, but such link may not represent any direct transmission relationship. The possibility of the presence of intermediate nodes cannot be ruled out, since the network cannot be a complete one comprising all HIV-infected persons, including undiagnosed ones. In implementing the study, we have included all HIV services in the territory of Hong Kong so as to narrow the gaps in multiple cascades or clusters. Yet, missing nodes in the transmission cascade are inevitable. Second, self-selection bias could be present and our results showed that almost 90% were MSM while during the study period, less than 80% of the newly diagnosed reported man-to-man sex as the transmission route [27]. The recruitment of a sizable number of MSM had nevertheless allowed us to stratify them and identify factors associated with a higher importance in the network and cascades. Third, although we adopted a self-administered questionnaire approach, social desirability bias could not be eliminated and may affect the accuracy of behavioural profiles. The plausible misclassification in risk factors may have weakened the significance of associations. Finally, recall bias could have an impact on the accuracy of behaviour-related items and estimated infection time. The latter was validated by the diagnosis date such that infection time precedes diagnosis. No directionality was drawn if such data was missing or invalid to minimize error.

In conclusion, transmission cascades extracted from transmission networks could provide useful information on the characteristics of transmission dynamics for molecular surveillance of HIV infection. Network and information diffusion metrics could help identifying factors contributing to onward transmission in molecular epidemiological studies. Our results could inform future prevention strategies planning, such as PrEP programme inclusion criteria and its promotional or delivery channels for MSM.

## Data Availability

The data for supporting the findings of this study are available from the respective collaborating hospitals and clinics. The researchers are not owners of the data. Restrictions apply to the availability of these data, which were used under data access approvals for this study. Li Ka Shing Institute of Health Sciences is acknowledged for providing technical support.
